# Development of a Nomogram to Predict the Risk of Chronic Active Epstein-Barr Virus Infection Progressing to Hemophagocytic Lymphohistiocytosis

**DOI:** 10.3389/fmed.2022.826080

**Published:** 2022-02-04

**Authors:** Xiaodan He, Jingshi Wang, Deli Song, Zhao Wang

**Affiliations:** Department of Hematology, Beijing Friendship Hospital, Capital Medical University, Beijing, China

**Keywords:** CAEBV, HLH, thrombocytopenia, alanine aminotransferase, cytopenia, nomogram

## Abstract

**Background:**

Chronic active Epstein-Barr virus infection (CAEBV) disease is sometimes associated with an aggressive clinical course, such as hemophagocytic lymphohistiocytosis (HLH). To explore the risk factors and predict the risk of CAEBV infection progressing to HLH, a retrospective research study was conducted.

**Methods:**

We retrospectively reviewed the medical records of 187 CAEBV-infected patients who were admitted to our center between January 2015 and December 2020. The patients were followed up until May 2021. The patients were divided into a progression-to-HLH group and a no-progression-to-HLH group. Demographic, clinical and laboratory data were collected for each patient.

**Results:**

Among the 121 CAEBV-infected patients who fulfilled the study's inclusion criteria, 48 (30.7%) patients did not progress to HLH, and 73 (60.3%) patients progressed to HLH. The median time from CAEBV infection to progression to HLH was 14 months, and the cumulative incidence rate of HLH increased as the duration of follow up increased (24.9, 47.3, 55.1, and 85.2% at 1, 3, 5, and 10 years, respectively). Multivariate analyses showed that the independent risk factors for CAEBV progression to HLH were plasma EBV-DNA load (OR = 3.239, 95% CI 1.219–8.603, *P* = 0.018), Platelet count (OR=0.991, 95%CI 0.985–0.998, *P* = 0.010), elevated alanine aminotransferase (OR=1.019, 95%CI 1.005–1.034, *P* = 0.009) and ≥2 of 3 lineages of cytopenia (OR=8.364, 95%CI 1.062–65.839, *P* = 0.044). The regression coefficients (β) from the multivariate logistic model were used to construct a model for estimating the risk of CAEBV infection progressing to HLH. The discriminatory ability of the model was good, and the area under the receiver operating characteristic (ROC) curve (AUC) was 0.925.

**Conclusion:**

plasma EBV-DNA load, platelet count, elevated alanine aminotransferase and ≥ 2 of 3 lineages of cytopenia increase the risk of CAEBV infection progressing to HLH. A nomogram can be used to estimate the risk of CAEBV-infected patients progressing to HLH.

## Introduction

Chronic active Epstein-Barr virus infection (CAEBV) disease is a lymphoproliferative disease associated with EBV infection that is characterized by chronic or recurrent infectious mononucleosis-like symptoms, including fever, lymphadenopathy, hepatitis, splenomegaly or pancytopenia (1, 2). The clinical course of CAEBV disease is heterogeneous: some patients may survive for more than 10 years without effective treatment, whereas others progress rapidly to hemophagocytic lymphohistiocytosis (HLH), multiple organ failure, or leukemia/lymphoma within a few years (3–6). HLH is a life-threatening syndrome involving excessive immune activation, and it is characterized by an inflammatory cytokine storm that causes multi-organ dysfunction. The clinical manifestations and laboratory findings characteristic of HLH include fever, hemocytopenia, splenomegaly, hypertriglyceridemia, hyperferritinemia, hypofibrinogenemia, and hemophagocytosis in the bone marrow, spleen, or lymph nodes (7, 8). The incidence of the progression of CAEBV infection to HLH is currently unknown. A Japanese study showed that 24.4% of CAEBV-infected patients progressed to HLH (9). In general, CAEBV infection is a fatal disease with high mortality and morbidity. Once CAEBV infection progresses to HLH, the prognosis worsens. Standard therapy regimens for CAEBV infection have not been established. Hematopoietic stem cell transplantation (HSCT) is currently the only regimen that can cure the disease (10, 11). However, HSCT presents significant risks and complications for patients. Although HSCT treatment of CAEBV infection may result in life-threatening complications, patients with poor prognosis require aggressive treatment to reduce or eliminate EBV-infected cells. Studies have shown that early HSCT for patients in relatively good clinical condition may improve the prognosis of HSCT (12); therefore, predicting the progression of CAEBV to HLH is particularly important for patients with a potential poor prognosis.

The purpose of this study was to explore the clinical and laboratory risk factors for the progression of CAEBV infection to HLH, which is extremely significant for enabling clinicians to adjust their treatment choices in a timely manner, thus prolonging the survival time and improving the prognosis of CAEBV-infected patients.

## Materials and Methods

### Study Design

A retrospective analysis was performed on 187 patients with a definitive diagnosis of CAEBV infection who were admitted to our center from January 2015 to December 2020. The patients were followed up until May, 2021. The median follow-up time of this study was 51 months. **Inclusion criteria:** 1) meeting the recently revised diagnostic criteria for CAEBV disease, including persistent infectious mononucleosis like symptoms for more than 3 months, increased EBV-DNA in peripheral blood, histological evidence of organ disease, and EBV-RNA or viral protein in affected tissues (13); 2) meeting the diagnostic criteria of HLH (for patients for whom CAEBV infection progressed to HLH) (7); and 3) complete laboratory examination results and case data. **Exclusion criteria:** 1) Presence of autoimmune or immunodeficiency diseases; 2) progression of CAEBV to neoplastic diseases, such as lymphoma, including extranodal NK/T cell lymphoma, aggressive NK cell leukemia, and peripheral T cell lymphoma; 3) Acute EBV-associated HLH; and 4) CAEBV patients received immunotherapy or allogeneic hematopoietic stem cell transplantation therapy. The 121 patients who met the inclusion criteria were divided into a progression-to-HLH group (73 cases) and a no-progression-to-HLH group (48 cases).

For patients no-progression-to-HLH, the blood drawn for analysis in this study were 1 week prior or after the diagnosis of CAEBV. While, for progression-to-HLH patients, the time points of blood drawn for analysis in the study were 1 week prior or after the diagnosis of CAEBV and HLH.

### Methods

The following data were collected for each patient: the age at onset, gender, clinical symptoms, interval time from clinical symptoms to diagnosis of CAEBV infection, EBV-infected lymphocyte subpopulations, EBV-DNA quantity in plasma, EBV-DNA quantity in peripheral blood mononuclear cells (PBMCs), splenomegaly, ≥2 of 3 lineages of cytopenia, platelet count (PLT), alanine aminotransferase (ALT), albumin (ALB), total bilirubin (TB), lactate dehydrogenase (LDH), decreased natural killer (NK) cell activity, hemophagocytosis in bone marrow, and presence of abnormal phenotypic cells in bone marrow. Real-time fluorescent quantitative PCR (qPCR) and TaqMan hydrolysis probes were used to detect EBV-DNA in plasma and PBMC. Intracellular EBV-DNA copies were quantified by qPCR in sorted B-, T-, and NK-cells. The clinical and laboratory data of the two groups were comparatively analyzed to explore the risk factors for the progression of CAEBV infection to HLH. The regression coefficients (β) from the multivariate logistic model were used to construct a model for estimating the risk of CAEBV infection progressing to HLH.

### Statistical Analysis

Categorical variables were compared using the chi-square test, and the Wilcoxon rank sum test of two-side test was applied to all continuous variables because the distributions of most of these variables were skewed. The medians and 25^th^-75^th^ percentiles of the continuous variables were presented. Categorical variables were presented as proportions. The cumulative incidence of HLH estimate using the Kaplan-Meier method. Logistic regression analysis was used for multivariate analysis. A value of *p* < 0.05 was considered statistically significant. The nomogram and time-dependent ROC curve were established with R (http://www.R-project.org) and EmpowerStats software (www.empowerstats.com, X&Y solutions, Inc. Boston MA). Other analyses were performed by IBM®SPSS® software, version 20.0 (IBM Corporation, Armonk, NY, USA).

## Results

### Epidemiology

**Figure 1:**
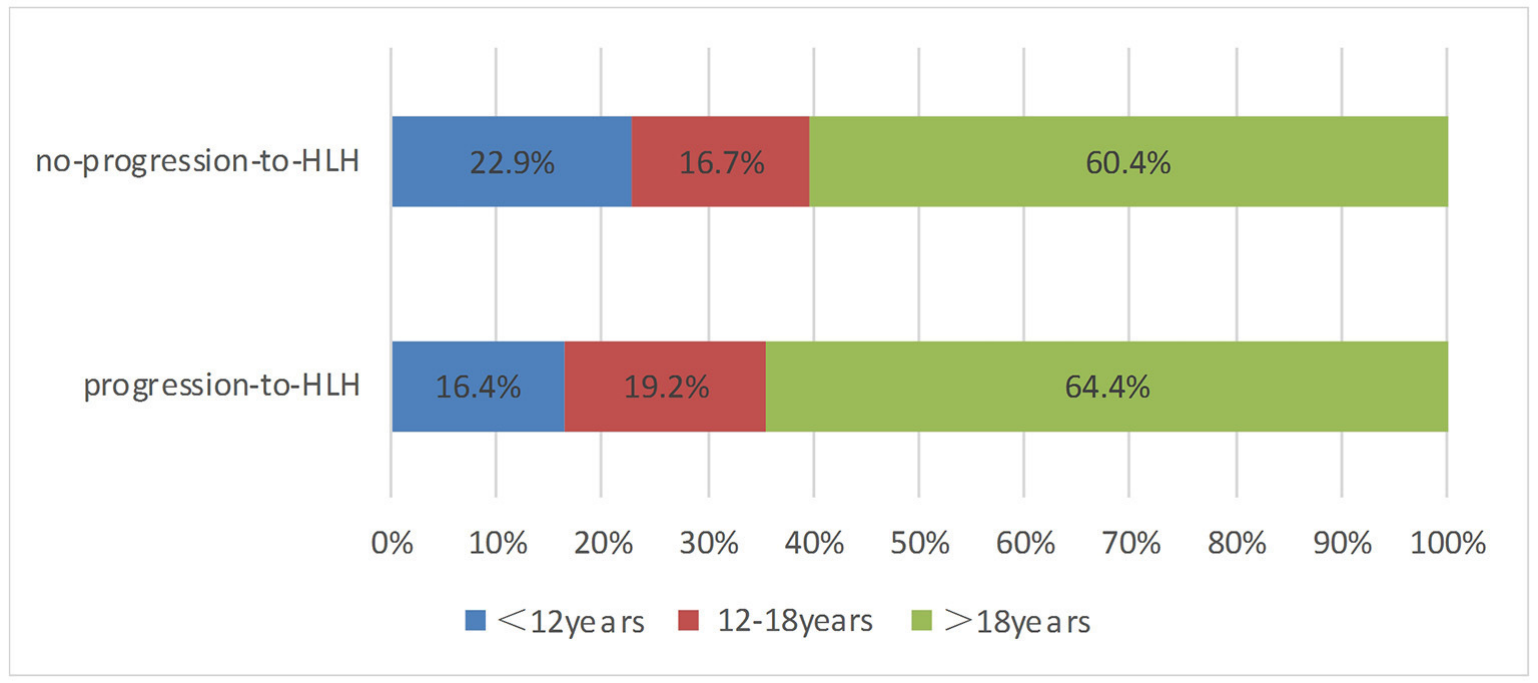
The age distribution of CAEBV infection progression to HLH and not progression to HLH.

**Figure 2:**
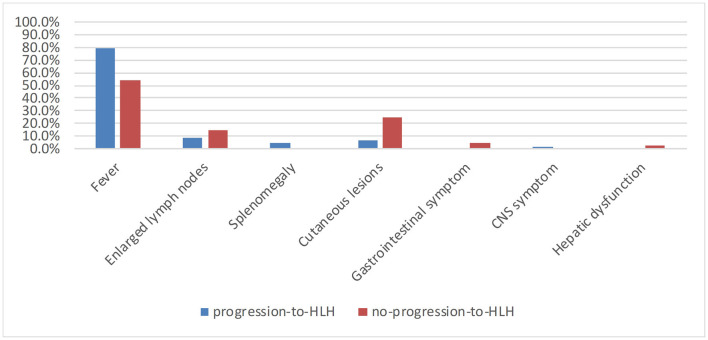
Symptoms and signs at onset of patients with CAEBV infection progression to HLH or not.

A total of 121 patients with CAEBV infection who meet the inclusion criteria were enrolled in this study, where 73 cases progressed to HLH and 48 cases did not progress to HLH. The age at the onset of disease ranged from 2 to 74 years (mean, 27 years), including 45 (37.2%) young people (under 18 years of age) and 76 (62.8%) adults. The age distribution of the two groups is shown in Figure 1. Among the 121 patients, 77 were male and 44 were female, with a male: female ratio of 1.75:1. Figure 2 shows the signs and symptoms of the two groups at the onset of CAEBV infection as percentages. Most patients presented with high fever in both groups (progression-to-HLH, 79.5%; no-progression-to-HLH, 54.2%); enlarged lymph nodes were found in 8.2% of the patients who progressed to HLH compared to 14.6% of the patients who did not progress to HLH; cutaneous lesions were present in 6.8% of the progression-to-HLH group and 25% of the no-progression-to-HLH group. Splenomegaly and central nervous system (CNS) symptoms were only presented by CAEBV-infected patients who progressed to HLH and was found in 4.1 and 1.4%, respectively, of these patients. Gastrointestinal symptoms (4.2%) and hepatic dysfunction (2.1%) were observed in patients who did not progress to HLH.

### Clinical and Laboratory Features

The 121 CAEBV-infected patients were divided into a progression-to-HLH group (*n* = 73) and no-progression-to-HLH group (*n* = 48), and the clinical and laboratory characteristics of the two groups were compared. Table 1 is a comparison of the laboratory data obtained at diagnosis for the two groups. Univariate analysis showed that the risk factors associated with the progression of CAEBV to HLH were the plasma EBV-DNA load (*P* < 0.001), the PBMC EBV-DNA load (*P* = 0.023), splenomegaly (*P* < 0.001), platelet count (*P* < 0.001), elevated alanine aminotransferase (*P* < 0.001), ≥2 of 3 lineages of cytopenia (hemoglobin <90 g/L, platelet count <100 × 10^9^/L, neutrophils count <1.0 × 10^9^/L) (*P* < 0.001), hypoalbuminemia (*P* < 0.001), elevated total bilirubin (*P* < 0.001), increased LDH (*P* < 0.001), hemophagocytosis in bone marrow (*P* < 0.001), abnormal phenotypic cells in bone marrow (*P* < 0.001) and D-dimer levels (*P* < 0.001). Factors for which no significant differences were observed between the two groups included gender, age, time interval from the onset of symptoms to the diagnosis of CAEBV, EBV-infected lymphocyte subpopulations, APTT, PT and low or absent NK cell activity (all, *P* > 0.05) (Table 1). Multivariate analysis [including the plasma and PBMC EBV-DNA loads, splenomegaly, platelet count, elevated alanine aminotransferase, ≥2 of 3 lineages of cytopenia, hypoalbuminemia, elevated total bilirubin, hemophagocytosis in bone marrow, abnormal phenotypic cells in bone marrow and D-dimer] showed that the independent factors for progression of CAEBV to HLH were plasma EBV-DNA load (OR = 3.239, 95% CI 1.219–8.603, *P* = 0.018), platelet count (OR = 0.991, 95% CI 0.985–0.998, *P* = 0.010), elevated alanine aminotransferase (OR = 1.019, 95% CI 1.005–1.034, *P* = 0.009) and ≥2 of 3 lineages of cytopenia (OR = 8.364, 95% CI 1.062–65.839, *P* = 0.044) (Table 2). Additionally, we found the best cut-off value for plasma EBV-DNA obtained from the ROC curve was 10^2.84^ copies/mL (AUC was 0.775, 95% CI 0.673–0.858, *P* < 0.001, sensitivity 0.932, specificity 0.524%).

**Table 1:** Univariate analysis of factors related with chronic active Epstein-Barr virus (EBV) infection progress to HLH [n(%) or M(P25 P75)].

**Factor**		**All patients**	**Whether CAEBV progressed to HLH or not**		***X^**2**^/Z***	***P*-value**
			**No (*n =* 48)**	**Yes (*n =* 73)**		
Gender	Male	77 (63.6)	32 (66.7)	45 (61.6)	0.316	0.574
	Female	44 (36.4)	16 (33.3)	28 (38.4)		
Age (years)	<12	23 (19.0)	11 (22.9)	12 (16.4)	0.812	0.666
	12–18	22 (18.2)	8 (16.7)	14 (19.2)		
	> 18	76 (62.8)	29 (60.4)	47 (64.4)		
Time intervals from onset symptoms to diagnosis of CAEBV (months)		10 (4.5~24)	12 (6~24)	9 (4~14)	1.396	0.163
EBV-infected	T	13 (13.5)	7 (53.8)	6 (46.2)	7.387	0.057
lymphocyte	NK	17 (18.1)	3 (17.6)	14 (82.4)		
subpopulations	B	7 (7.45)	5 (71.4)	2 (28.6)		
	≥ 2 types	59 (62.8)	23 (39.0)	36 (61.0)		
EBV-DNA load in plasma (log copies/mL)		3.72 (2.70~4.37)	2.82 (2.70~3.93)	4.19 (3.52~4.78)	4.421	<0.001
EBV-DNA load in PBMC (log copies/mL)		4.76 (3.67~5.98)	3.93 (3.17~5.45)	5.07 (3.81~6.09)	2.270	0.023
Splenomegaly		90 (74.4)	28 (58.3)	62 (84.9)	10.751	<0.001
≥ 2 of 3 lineages cytopenia		82 (67.8)	21 (43.8)	61 (83.6)	21.013	<0.001
Platelet count (10^9^/L)		155 (80~236.5)	236.5 (189.25~332.75)	96 (60~163.5)	6.154	<0.001
Alanine aminotransferase (U/L)		53 (21~123.5)	22 (16~54.5)	78 (38~158)	4.981	<0.001
Albumin (g/L)		34.2 (29.4~39.93)	38.7 (33.8~43)	31 (25.6~36.8)	4.494	<0.001
Total bilirubin (umol/L)		16.01 (9.43~26.38)	10.56 (7.22~20.39)	18.09 (11.65~45.02)	4.027	<0.001
Lactate dehydrogenase (U/L)		345 (207~499)	226 (185~394)	409.5 (287.5~701.5)	4.341	<0.001
Absent NK-cell activity		48 (42.1)	18 (40.9)	30 (42.9)	0.042	0.838
Hemophagocytosis		26 (21.5)	4 (8.3)	22 (30.1)	6.919	0.009
Abnormal phenotypic cells		29 (24.0)	6 (12.5)	23 (31.5)	5.741	0.017
PT(s)		11.9 (11~12.8)	11.8 (11.1~12.4)	12.2 (10.9~13.45)	1.134	0.257
APTT(s)		32.1 (27.95~36.6)	31.3 (27.7~34)	32.6 (27.9~38.7)	1.401	0.161
D-dimer (mg/L)		0.9 (0.5~2.07)	0.6 (0.4~0.95)	1.7 (0.7~3)	4.809	<0.001

**Table 2:** The independent risk factors for CAEBV infection progression to HLH.

**Factor**	**B**	**SE**	**Walds**	**df**	***P*-value**	**OR**	**95% CI**
EBV-DNA quantity in plasma (log copies/mL)	1.175	0.498	5.558	1	0.018	3.239	1.219~8.603
Platelet count	−0.09	0.003	6.597	1	0.010	0.991	0.985~0.998
Alanine aminotransferase	0.019	0.007	6.788	1	0.009	1.019	1.005~1.034
≥ 2 of 3 lineages cytopenia	2.124	1.053	4.067	1	0.044	8.364	1.062~65.893

### Treatment Protocols of the Two Groups After Diagnosis of CAEBV

Of the 121 patients diagnosed with CAEBV, 39 patients did not receive any treatment, and 18 of them progressed to HLH; 22 patients were treated with antiviral therapy, and 20 patients developed HLH; 31 patients were treated with methylprednisolone or dexamethasone, and 27 patients developed HLH; 2 patients were treated with ruxolitinib, and 1 patient progressed to HLH; 1 patient was treated with rituximab without progression to HLH by the end of follow-up; 3 patients were treated with asparaginase, and 2 patients progressed to HLH; 7 patients were treated with HLH-94 or HLH-2004 regimen, and 1 patient progressed to HLH; 16 patients were treated with DEP regimen (Liposomal doxorubicin + etoposide + methylprednisolone), ruxolitinib + DEP regimen, or asparaginase + DEP regimen, and 4 patients progressed to HLH.

### Treatment Protocols After CAEBV Progression to HLH

There were 73 patients diagnosed with CAEBV progression to HLH. Fourty-six patients adopted DEP regimen, ruxolitinib + DEP regimen, or asparaginase + DEP regimen as initial induction treatment, of which 21 patients underwent allogeneic hematopoietic stem cell transplantation (allo-HSCT) followed DEP regimen and 10 patients died by the end of follow-up; 25 patients were not able to undergo allo-HSCT for various reasons, and 7 patients died by the end of follow-up. Nine patients were treated with HLH-94 or HLH-2004 regimen initially, of which 5 patients were treated with DEP regimen as salvage therapy, and 3 patients underwent allo-HSCT were alive by the end of follow-up; 6 patients failed to undergo allo-HSCT, and 3 patients died by the end of follow-up. Four patients adopted E-CHOP like regimens (etoposide + cyclophosphamide + epriubicin + vincristine + glucocorticosteroid) or E-COP like regimens (etoposide + cyclophosphamide + vincristine + glucocorticosteroid) as initial treatment, 1 patient died after allo-HSCT, and 2 patients died without undergoing allo-HSCT. Six patients were treated with ruxolitinib or combined with methylprednisolone, and 1 patient died. There were 7 patients' treatment protocols unclear, and 3 patients died at the end of follow-up. One patient treated with FC therapy (fludarabine + cyclophosphamide) was still alive at the end of follow-up.

### The Nomogram and Its Predictive Performance

**Figure 3:**
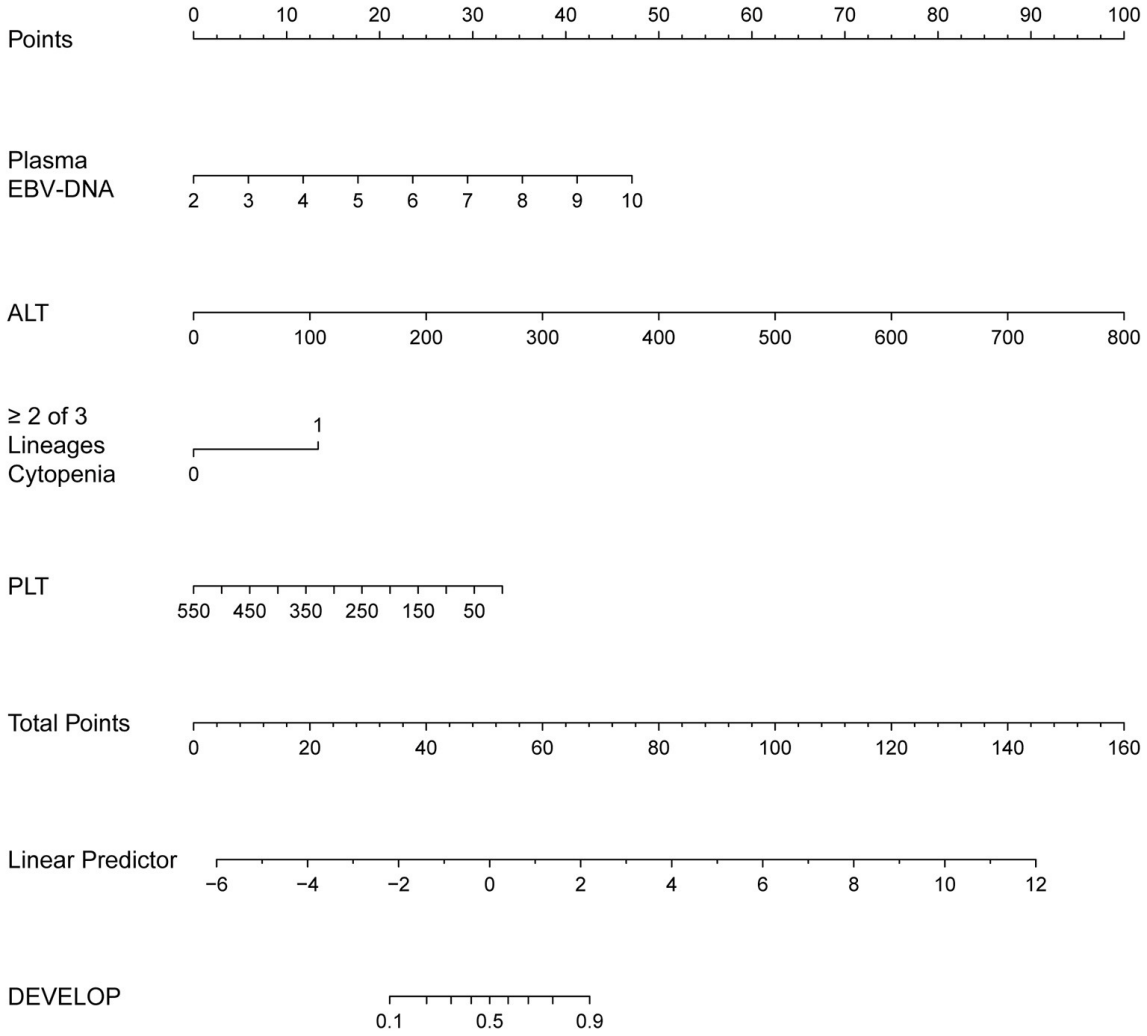
The nomogram to estimate the risk of CAEBV infection progress to HLH. To use the nomogram, find the position of each variable on the corresponding axis, draw a line to the points axis for the number of points, add the points from all of the variables, and draw a line from the total points axis to determine the probabilities of CAEBV infection progressed to HLH at the lower line of the nomogram.

**Figure 4:**
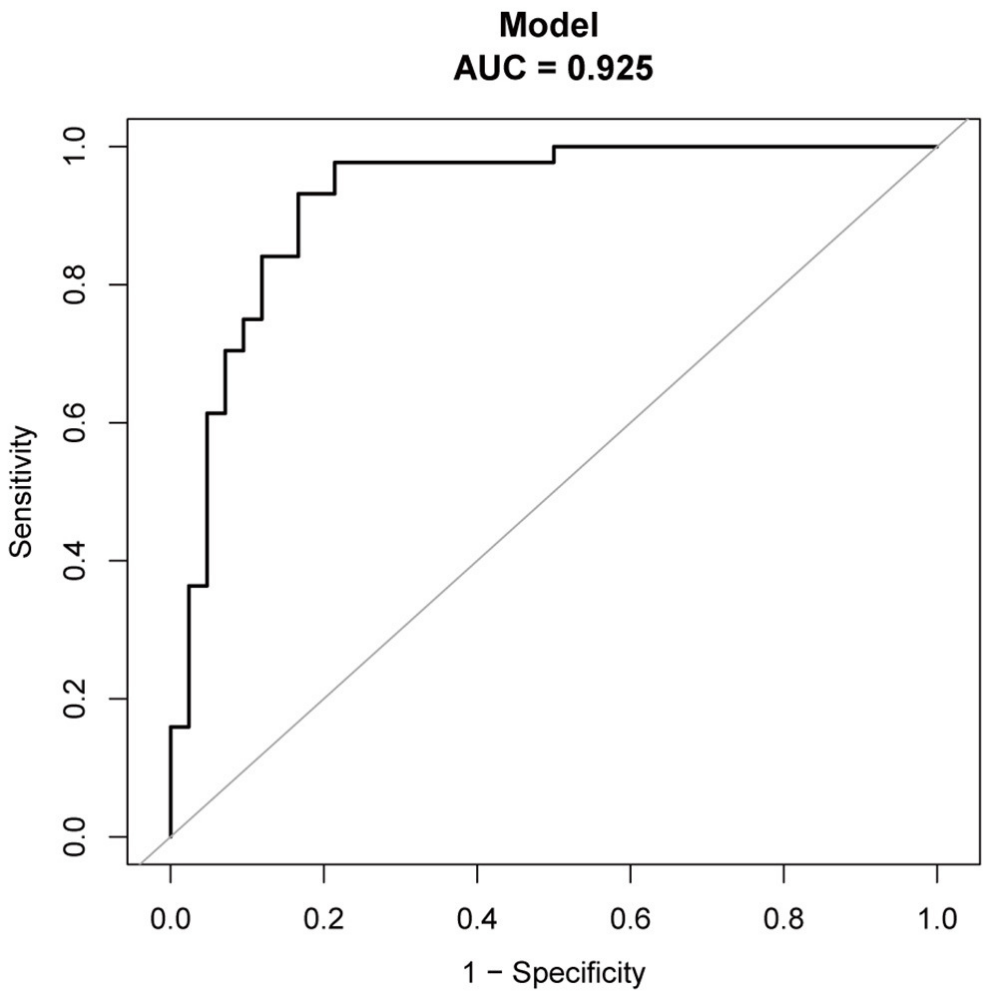
The AUC of the model from observed data (nomogram) was 0.925.

The regression coefficients (β) obtained using the multivariate logistic model were used to construct a model for estimating the risk of CAEBV infection progressing to HLH (Figure 3). The scoring model was as follows:−3.77 + 0.75^*^EBV-DNA load + 0.02^*^ elevated alanine aminotransferase + 1.71^*^ (≥2 of 3 lineages of cytopenia) - 0.01^*^ platelet count. The performance of the nomogram was measured by ROC curves, and the AUC for the model was found to be 0.925 (95% CI 0.867–0.984) using the observed data. The cut-off score was−0.271 with a sensitivity of 0.932 and a specificity of 0.833 (Figure 4).

### Time Interval for CAEBV to Progress to HLH

**Figure 5:**
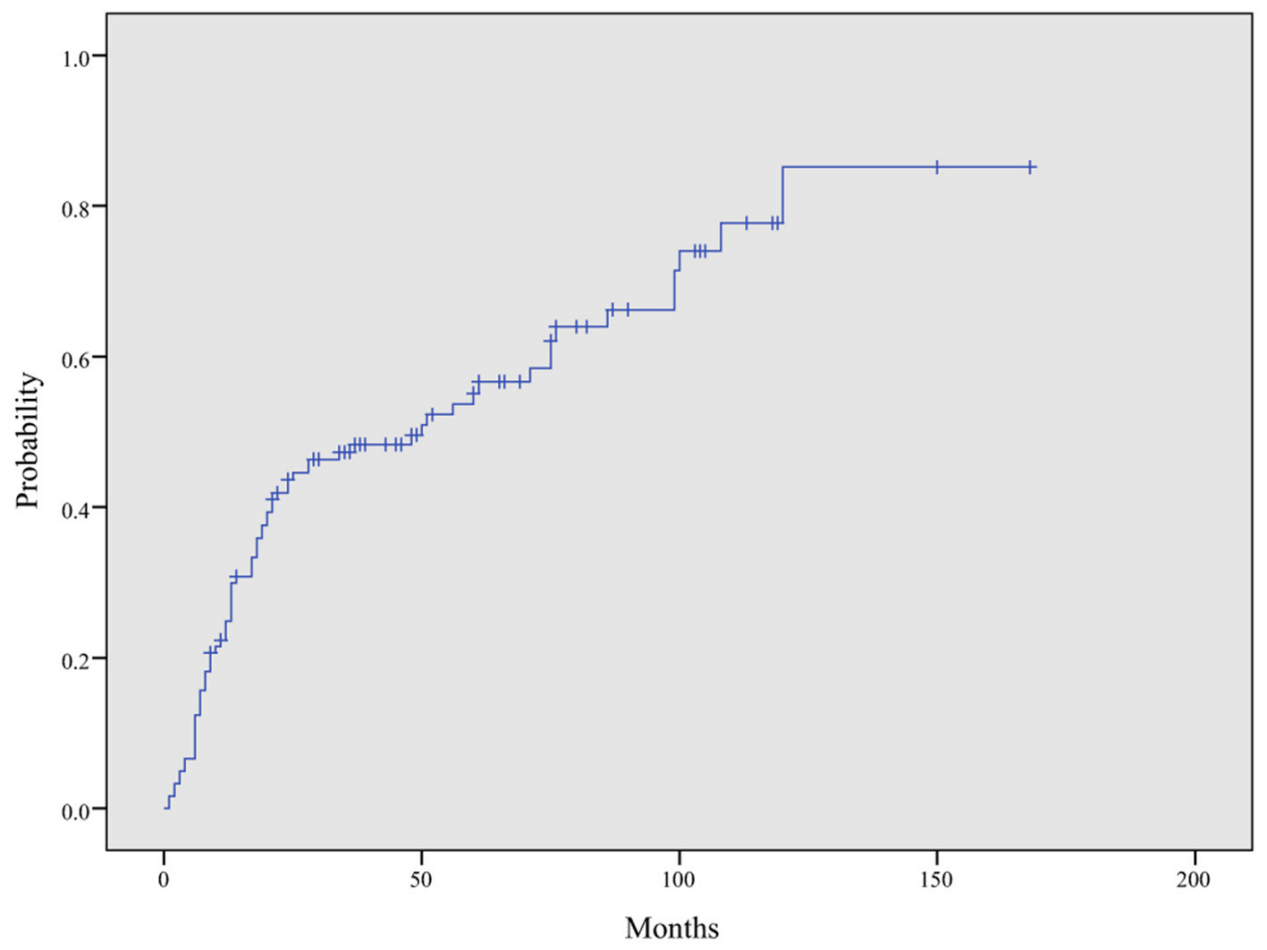
Cumulative incidence curve of HLH in CAEBV patients. The cumulative incidence of HLH at 1, 3, 5, and 10 years was 24.9, 47.3, 55.1, and 85.2%, respectively, by Kaplan–Meier analysis.

The time from CAEBV infection progression to HLH was in the range of 1–120 months with a median progression time of 14 months. The cumulative incidence rate of HLH increased as the duration of follow up increased (24.9, 47.3, 55.1, and 85.2% at 1, 3,5, and 10 years, respectively) (Figure 5). Among patients under 18 years of age, 26 (21.5%) developed HLH, with a median progression time of 20 months. However, in patients over 18 years old, 47 cases (38.8%) progressed to HLH, with a median progression time of 13 months. The median progression times of the two groups were not significantly different (*P* = 0.333).

## Discussion

CAEBV infection is considered to be a fatal disease. Approximately 50% of CAEBV-infected patients die within 5 years of diagnosis due to progression to lymphoma, HLH, heart or liver failure. Almost all patients who do not receive reasonable and effective treatment die within 15 years of diagnosis due to various complications, including HLH, multiple organ failure, or leukemia/lymphoma (14). The risk factors that predict CAEBV progression to HLH are significant for enabling clinicians to adjust therapies in a timely manner to improve prognosis.

This study is the first analysis of the risk factors for the progression of CAEBV infection to HLH. We retrospectively reviewed the medical records of 187 CAEBV-infected patients who were admitted to our hospital between January 2015 and December 2020, where 121 patients met the inclusion criteria for the study. Four factors were identified as independent risk indicators for the progression of CAEBV infection to HLH, including plasma EBV-DNA load, platelet count, elevated alanine aminotransferase and ≥2 of 3 lineages of cytopenia. Previous studies have found several of these variables to be risk factors for the prognosis of CAEBV infection. Hiroshi Kimura et al. found that platelet count, late onset of disease and T-cell infection were correlated with CAEBV patient mortality (9). LU Gen and his colleagues showed that platelet count and decreases in albumin are potential risk factors for a poor prognosis of CAEBV infection (15). Hiroshi Kimura et al. revealed that age at onset of disease (> 8 years) and liver dysfunction were risk factors for mortality, whereas transplant patients had a better prognosis (16). Interestingly, from our date, we find these risk factors related to the prognosis of CAEBV infection in previous studies were also indicators for CAEBV progression to HLH. These results indicate that CAEBV progress to HLH may has a worse prognosis.

It is noteworthy that CAEBV infection in patients with a persistent high EBV-DNA load and chronic unregulated active EBV replication are associated with poor prognosis. Hiroshi Kimura et al. studied 30 CAEBV-infected patients and found that all patients had high viral loads in their peripheral blood (more than 10^2.5^ copies/μg DNA) (9). LU Gen et al. studied 53 Chinese CAEBV pediatric patients and found a mean plasma EBV DNA level of 10^3.7^ copies/mL for 23 cases (15). Akihiko Maeda et al. examined the relationship between the clinical manifestation of CAEBV and EBV-DNA load and found that fever was correlated with the virus load (12). In the present study, we also found that the plasma EBV-DNA load was an independent risk factor for the progression of CAEBV to HLH. A ROC curve analysis showed that the best cut-off value for plasma EBV-DNA was 10^2.84^ copies/ml, indicating that the patients with CAEBV infection are prone to HLH, even though plasma EBV-DNA is low. The EBV-DNA load in PBMCs, however, was found not the predictor of CAEBV progression to HLH. This maybe EBV in plasma had higher specificity and sensitivity for EBV infection related diseases compared with EBV in PBMCs (16).

In previous studies, EBV infection of T cells was found to be more likely to progress to multiple organ failure (MODS) and had a worse prognosis than EBV infection of other lymphocyte subpopulations (9, 17). However, in the present study, the number of EBV infection of T, B, NK lymphocyte subpopulations and ≥2 lineages lymphocyte subpopulations were 6 (46.2%), 2 (28.6%), 14 (82.4%), and 36 (61.0%), respectively, in the progression-to-HLH group and 7 (53.8%), 5 (71.4%), 3 (17.6), and 23 (39.0%), respectively, in the no-progression-to-HLH group. We did not find that EBV infection of T cell subpopulations was more likely to progress to HLH than EBV infection of other lymphocyte subpopulations. This result may have been obtained because some patients were infected with B and T/NK lymphocyte subpopulations at the same time, where the main infection was from B lymphocyte subpopulations. The clinical course of EBV infection of B lymphocyte subpopulations was not aggressive and associated with a better prognosis than for other lymphocyte subpopulations (18).

Previous reports showed a better prognosis for CAEBV infection for children than adults. Arai et al. showed more progressive and aggressive courses in adult-onset CAEBV patients than childhood-onset patients (19). A prospective study conducted by Hiroshi Kimura et al. showed that an older onset age (≥8 years) was associated with mortality in CAEBV patients (9, 17). However, a study of 53 Chinese pediatric patients with CAEBV infection conducted by LU Gen and his colleagues revealed a severe clinical course and poor prognosis (15). In the present study, we found no statistically significant association between the age of onset and the progression of CAEBV to HLH (*P* = 0.666). Additionally, the median progression times for patients under 18 years of age and over 18 years old were also not significantly different (*P* = 0.333). Ayako Arai et al. reviewed 23 adult-onset CAEBV infection patients, showed that the time during from the onset of disease to initiation treatment averaged 20 months and 7 patient died at an average of 8 months after initiation of treatment (19). Kimura et al. reviewed 30 Japanese CAEBV infection patients, found that young patients could have a time duration without treatment of 12–336 months (mean 71 months) and the 5 year survival rate was 0.68 ± 0.06 (20). In our study, the median time for CAEBV infection to progress to HLH was 14 months, and the cumulative incidence rate of HLH increased as the duration of follow up increased (24.9, 47.3, 55.1, and 85.2% at 1, 3, 5, and 10 years, respectively), which indicating that the clinical course of CAEBV is rapidly progressive and aggressive.

In addition, we developed a nomogram that is easy to use and integrates 4 predictors for the risk of CAEBV-infected patients progressing to HLH. The nomogram showed good predictive accuracy based on an AUC of 0.925 and can be easily used by clinicians. The four variables required for the nomogram are generally readily available at admission. These indicators can also be monitored during the clinical course of the disease to predict the risk of progression of CAEBV to HLH. To improve the therapeutic effect and prognosis of CAEBV, aggressive treatment, such as HSCT, could be considered once the prediction of progression to HLH is high. In general, this nomogram serves as a reference for clinicians to predict the risk of progression of CAEBV infection to HLH. The decision to undertake aggressive treatment also depends on a patient's condition, willingness, and finances.

In general, it is extremely important to explore the clinical and laboratory risk factors for CAEBV progressing to HLH. As this retrospective study was conducted in a single center using a small sample size, a prospective study should be conducted in multicenters on large populations of CAEBV-infected patients. Other parameters, such as lymphocyte subpopulations values, immunoglobulins levels, and soluble CD25 etc. should be discussed in the next prospective study.

## Conclusion

In conclusion, 4 factors were identified in this study as independent risk indicators for the progression of CAEBV infection to HLH, including an increased plasma EBV-DNA load, platelet count, elevated alanine aminotransferase and ≥2 of 3 lineages of cytopenia. A nomogram can be used to estimate the risk of patients with CAEBV progressing to HLH.

## Data Availability Statement

The raw data supporting the conclusions of this article will be made available by the authors, without undue reservation.

## Ethics Statement

The studies involving human participants were reviewed and approved by the Ethics Committee of the Beijing Friendship Hospital. Written informed consent from the participants' legal guardian/next of kin was not required to participate in this study in accordance with the national legislation and the institutional requirements.

## Author Contributions

XH designed and performed the research and wrote the paper. ZW designed the research and supervised the report. JW provided clinical advice and supervised the report. DS contributed to the data collection and statistical analysis. All authors approved the final manuscript.

## Funding

This work was supported by grants from the National Natural Science Foundation of China (Grant No. 81871633), the Beijing Natural Science Foundation (Grant No. 7181003), Beijing Municipal Administration of Hospitals' Ascent Plan (DFL20180101).

## Conflict of Interest

The authors declare that the research was conducted in the absence of any commercial or financial relationships that could be construed as a potential conflict of interest.

## Publisher's Note

All claims expressed in this article are solely those of the authors and do not necessarily represent those of their affiliated organizations, or those of the publisher, the editors and the reviewers. Any product that may be evaluated in this article, or claim that may be made by its manufacturer, is not guaranteed or endorsed by the publisher.
